# Calcineurin regulates morphological development, stress responses and virulence in *Fonsecaea monophora*

**DOI:** 10.1371/journal.pntd.0013816

**Published:** 2025-12-10

**Authors:** Minying Li, Huan Huang, Dongmei Li, Judun Zheng, Yinghui Liu, Yangxia Chen, Zhenmou Xie, Liyan Xi, Hongfang Liu

**Affiliations:** 1 Dermatology Hospital, Southern Medical University, Guangzhou, China; 2 Department of Microbiology-Immunology, Georgetown University Medical Center, Washington, District of Columbia, United States of America,; 3 Department of Dermatology, Sun Yat-sen Memorial Hospital, Sun Yat-Sen University, Guangzhou, China; FIOCRUZ: Fundacao Oswaldo Cruz, BRAZIL

## Abstract

*Fonsecaea monophora* is a major cause of chromoblastomycosis (CBM) in southern China. While calcineurin is known to be a virulence factor in several fungi, its role in *F. monophora* remains poorly understood. In this study, we characterized the function of calcineurin in *F. monophora* by examining mutants of the two calcineurin subunit genes (*cnaA* and *cnaB*). The mutants exhibited significant defects in conidiation, germination, morphogenesis (including the transformation into muriform cells), resistance to various stressors, and increased susceptibility to triazole drugs. Importantly, the mutants showed greater susceptibility to macrophage-mediated killing in vitro and reduced virulence in a mouse model. Interestingly, deletion of the potential transcription factor gene (*crzA*) did not produce similar phenotypic changes, suggesting that calcineurin regulates these processes maybe independently of *crzA*. Our findings advance the understanding of calcineurin’s role in the morphology, antifungal resistance, and virulence of *F. monophora*. Given that the combination of itraconazole and tacrolimus has a synergistic effect on clinical strains, we propose that targeting calcineurin in combination with itraconazole may offer an effective therapeutic approach for CBM.

## Author summary

*F. monophora* is a predominant pathogen of chromoblastomycosis (CBM), a neglected but difficult-to-treat fungal infection. The calcineurin signaling pathway is a crucial regulator of virulence in many fungi, yet its role in *F. monophora* remained elusive. Our study reveals that calcineurin is a master regulator of this pathogen’s biology. Disrupting its function led to severe defects in fungal reproduction, morphological development (including the critical transition to tissue-invasive muriform cells), and resilience against oxidative and cell wall stresses. Consequently, these compromised mutants were more efficiently cleared by macrophages and exhibited profoundly attenuated virulence in a mouse model. Intriguingly, these pivotal functions operate independently of the canonical transcription factor *crzA*, suggesting a novel signaling mechanism. Furthermore, calcineurin impairment synergized with triazole antifungal drugs. Given the observed synergy between the calcineurin inhibitor tacrolimus and itraconazole against clinical strains, our work proposes targeted inhibition of calcineurin as a promising strategy to enhance the efficacy of conventional antifungal therapy for CBM.

## 1. Introduction

Chromoblastomycosis (CBM) is a chronic granulomatous fungal infection, commonly caused by the inoculation of various dematiaceous fungi into the skin and subcutaneous tissue. Treatment of CBM presents significant challenges due to its lengthy duration, high cost, frequent recurrence, and generally low cure rates [1]. If left untreated, CBM can progress to fibrosis or lead to other complications, resulting in disability and disfigurement that seriously impact patients’ quality of life. Due to its global prevalence (particularly among impoverished populations) and the difficulty in treatment, CBM was classified as a neglected tropical disease by the World Health Organization in 2017 [2]. Currently, no standardized therapy exists for CBM in clinical practice [3], and the mechanisms underlying its antifungal resistance remain poorly understood. In southern China (e.g., Guangdong), *F. monophora* is the predominant pathogen responsible for CBM [4]. This fungus transforms into parasitic sclerotic bodies within the host, a process linked to both virulence and antifungal drug resistance, which enables it to effectively evade host defenses [5]. At present, the pathogenic mechanisms of *F. monophora* are not well characterized, and there is an urgent need to identify the relevant virulence factors that contribute to its pathogenicity and treatment resistance.

In recent years, the calcineurin signaling pathway has been extensively studied in eukaryotes. This pathway transduces calcium ion signals into cellular responses through calmodulin-binding proteins (including calcineurin), calmodulin-dependent protein kinases, and histone deacetylases [6]. Calcium ion concentrations within the cytoplasm are regulated by calcium pumps or Ca² ⁺ channels [7], alongside sensor proteins like calmodulin (CaM). CaM is a primary sensor for cytoplasmic calcium ions and a trigger for downstream cellular responses. Calcineurin (CN), also known as protein phosphatase 2B (PP2B), is a calcium/calmodulin-activated serine-threonine phosphatase consisting of two subunits, A and B. In this heterodimeric phosphatase, the A subunit provides catalytic activity and contains three regulatory domains: the CNB subunit binding site, the calmodulin-binding domain, and the autoinhibitory domain. The B subunit binds calcium through four calcium-binding EF-hand motifs [8]. In the Ca² ⁺ -calcineurin (CaN) signaling pathway, calcineurin functions by dephosphorylating target proteins [9], enabling eukaryotic cells to perceive and adapt rapidly to diverse environmental conditions. Calcineurin in fungi is implicated in several fundamental processes, including cell growth, maintenance of cell wall integrity, and stress response [10].

Calcineurin signaling is also crucial for fungal virulence, enabling pathogen invasion and environmental adaptation through mechanisms such as the formation of infectious structures and modulation of host interactions [11]. Upon activation, calcineurin dephosphorylates and activates the transcription factor *crzA*, which is essential for cell survival and calcium homeostasis in certain yeasts [12,13]. This pathway is further associated with antifungal tolerance and cellular morphogenesis [14–16]. Notably, calcium has been shown to critically regulate the transition between hyphal and sclerotic body forms in chromoblastomycosis fungi *in vitro* [17], suggesting a potential role for the calcineurin pathway in the morphological transformation of *F. monophora* sclerotic bodies.

In *F. monophora*, no studies have yet investigated the role of the calcineurin pathway, and the specific mechanism by which it regulates virulence remains unclear. To address this, we used *Agrobacterium*-mediated transformation (ATMT) to knock out the calcineurin A subunit (*cnaA*, AYO21_04569) and B subunit (*cnaB*, AYO21_11439) in *F. monophora* (CBS269.37), respectively, generating calcineurin-deficient strains. We then assessed the resistance and toxicity responses of these knockout strains to various environmental factors compared with the wild type. Additionally, we created a putative *crzA* (AYO21_03504) knockout strain to investigate whether calcineurin’s regulatory effects in *F. monophora* depend on *crzA*. To our knowledge, this is the first study involving calcineurin gene knockout in *F. monophora*, which may provide insights into the pathological mechanism of CBM.

## 2. Materials and methods

### 2.1. Ethics statement

All animal experiments performed in this study were approved by the Experimental animal Ethics Committee of Guangdong Huawei Detection Co., LTD (202209001, animal license number **SCXK (Yue) 2023–0059**).

### 2.2. Strains, growth conditions, targeted gene deletion, and complementation analysis

Strains and plasmids used in this study are listed in **S1 Table**. *F. monophora* (CBS269.37) was used as the wild-type (WT) strain in all experiments. CBS 269.37 was obtained from the Westerdijk Fungal Biodiversity Institute (formerly CBS-KNAW). Transformation was performed using the *Agrobacterium* tumefaciens-mediated transformation (ATMT) method [18]. Plasmid construction and transformation experiments were similar to those in previous studies [19]. The *ΔcnaA*, *ΔcnaB* and *ΔcrzA* mutant strains were generated by transforming the WT with plasmids to delete the whole gene, followed by selection with hygromycin. The complemented *ΔcnaA::cnaA* and *ΔcnaB::cnaB* strain were generated by transforming *cnaA*-C-neo-pBHt2 and *cnaB*-C-neo-pBHt2 plasmid and selecting for geneticin-resistant transformants. The plasmids used for *cnaA, cnaB* and *crzA* targeted deletion (*cnaA*-hph-pBHt2, *cnaB*-hph-pBHt2 and *crzA*-hph-pBHt2) and complementation (*cnaA*-C-neo-pBHt2 and *cnaB*-C-neo-pBHt2) were constructed by In-Fusion HD Cloning Kit (Takara, Japan) according to the manufacturer’s instructions.

### 2.3. RNA isolation and real time PCR

Total RNA was extracted from each sample using TRIzol Reagent (Invitrogen, USA). Subsequently, the RNA was reverse transcribed into cDNA using the RevertAid First Strand cDNA Synthesis Kit (Thermo Scientific, USA; catalog no. #K1622). Quantitative real-time PCR (qRT-PCR) was performed on a Bio-Rad CFX96 Touch system (USA) with PowerUp SYBR Green Master Mix (Thermo Scientific, USA). The sequences of all primers used are listed in **S2 Table**.

### 2.4. Microscopy

All strains were inoculated onto PDA blocks covered with sterile coverslips and cultured at room temperature for 14 days. To examine morphogenesis, conidial germination, and cell wall architecture, the coverslips were stained with Lactophenol Cotton Blue and observed under a microscope. For scanning electron microscopy (SEM) and transmission electron microscopy (TEM) analyses, fungal samples were fixed in 2.5% glutaraldehyde at 4 °C for 24 hours. The samples were then washed six times with 0.1 M phosphate-buffered saline (PBS; 30 min per wash), post-fixed with 1% osmium tetroxide for 2 hours, and rinsed three times with PBS (10 min each). Dehydration was carried out through a graded ethanol series (30%, 50%, 70%, 80%, 90%, and 100%), followed by sequential immersion in a 1:1 (v/v) mixture of 100% ethanol and 100% acetone, and finally pure acetone (10 min per step). The dehydrated samples were embedded in resin. Ultrathin sections were prepared and examined using a transmission electron microscope. For SEM, after fixation and dehydration (as described above), the samples were transferred to isoamyl acetate, subjected to critical-point drying for at least 8 hours, sputter-coated with gold, and observed under a scanning electron microscope.

### 2.5. Antifungal susceptibility testing

To ensure standardized and reproducible inoculation, spores were used in all experiments. Antifungal susceptibility testing was conducted using the YeastOne (Thermo Fisher Scientific) or according to previously reported methods [20]. Conidia were suspended in RPMI 1640 medium at 1-2.5 × 10^4^ cells/mL. Terbinafine (TER) was dissolved in DMSO and diluted in RPMI 1640. The final concentrations of itraconazole, terbinafine and amphotericin B were ranged from 0.06 to 64 μg/mL. The fungal suspension-compound mixtures were incubated at 35 °C for 5 days. The minimum inhibitory concentration (MIC) was determined as the lowest drug concentration that completely inhibited macroscopic fungal growth.

### 2.6. *In vitro* drug interaction assay

Drug interaction experiments were conducted according to CLSI (American Clinical Laboratory Standards Institute) document M38-A3, using RPMI 1640 medium (pH 7.0) buffered with sodium triazomorphine propionate and sterilized by filtration. Spores were adjusted to (1–5) ×10^6^ CFU/mL with a hemocytometer, then diluted 100-fold to reach final inoculation concentration as (1 ~ 5) ×10^4^ CFU/mL. Test drugs (itraconazole, terbinafine, tacrolimus (Solarbio, Beijing)) were added to a 96-well plate at double concentrations (final concentrations: 0.015-2 mg/mL for itraconazole and terbinafine, 0.125-8 mg/mL for tacrolimus). Each plate contained growth and negative controls and were incubated at 35 °C for 5–7 days. The MIC was defined as the lowest concentration causing 100% inhibition of fungal growth. Drug interactions were classified using the fractional inhibitory concentration index (FICI): synergy (FICI ≤ 0.5), no interaction (0.5 < FICI ≤ 4.0), or antagonism (FICI > 4.0). Clinical isolates were sourced from the Dermatology Hospital of Southern Medical University

### 2.7. Fungal growth and stress resistance assays

The growth rate and spore production of mutants and wild-type (WT) strains were assessed on PDA medium at 26 °C and 37 °C for 14 days. Radial growth was measured every 3 days. For each strain, 5 µL of conidial suspension (1 × 10⁶ conidia/mL in PBS) was inoculated, and spore counts per mm² were determined after 14 days. Stress response was evaluated by inoculating 5 µL of conidial suspensions at various concentrations (10⁸ to 10⁴ conidia/mL) on PDA supplemented with 1.3 M KCl or NaCl (salt stress), 2.5 mM H₂O₂ (oxidative stress), 1.3 M sorbitol (osmotic stress), 100 µg/mL CFW (Calcofluor white), and Congo red (cell wall stress). Cultures were incubated for 14 days at 26 °Cand 37 °C. Based on previous research [19], the concentrations of KCl, NaCl, H_2_O_2_, CFW, Congo red, and sorbitol were selected.

### 2.8. Evaluation of macrophage killing ability

RAW264.7 macrophages were infected with *F. monophora* conidia at a multiplicity of infection (MOI) of 10 and incubated for 24 h at 37 °C in 5% CO₂. Subsequently, the macrophages were lysed with sterile water, and the released spores were serially diluted and plated on PDA. Plates were incubated at 25 °C for 7 days, and the number of colony-forming units (CFUs) was quantified. For TEM analysis, infected macrophages were fixed in a mixture of 4% paraformaldehyde and 2.5% glutaraldehyde, washed with PBS, post-fixed with 1% osmium tetroxide, dehydrated through a graded series of ethanol and acetone, embedded in resin, and examined using a JEM-1400 PLUS transmission electron microscope.

### 2.9. Galleria mellonella infection model

*Galleria mellonella* larvae infections were performed as previously described [21]. Healthy larvae weighing 250–300 mg were selected. Each larva was injected with 20 µL of PBS (control) or a conidial suspension (1 × 10⁵ conidia in PBS) into the last left proleg using an insulin syringe. The inoculated larvae were then incubated in the dark at 37 °C. At designated time points, larvae were randomly selected for fixation and hematoxylin and eosin (H&E) staining or for homogenization. Fungal burden was assessed by quantifying CFUs from the homogenized tissues.

### 2.10. Animal model

This study was carried out in strict accordance with the recommendations in the Guide for the Care and Use of Laboratory Animals of the Ministry of Science and Technology of the People’s Republic of China. BALB/c was purchased from Guangzhou Ruige Biological Technology Co. Ltd (Guangzhou, China). Female BALB/c mice (6–8 weeks old, 12 mice per group, totaling 48 mice) were randomly divided into four groups: control, wild-type strain, *cnaA* mutant and *cnaB* mutant. The mice were anesthetized by inhalation of 2-2.5% isoflurane (RWD Life Science, Cat# R510-22) mixed with oxygen using a small animal anesthesia system (AA-500, Guangzhou Biolight Biotechnology Co., Ltd.). Anesthesia was maintained with 1.5-1.8% isoflurane in oxygen mixture. The depth of anesthesia was monitored through foot pinch reflex and respiratory rate, which was maintained at 45–55 breaths per minute. Body temperature was kept at 37 °C using a heating pad. The footpad was injected with 50 µL of the fungal solution containing 1 × 10^6^ fungal cells (conidia) or PBS as the control into the plantar region. At 3, 7, and 14 days after injection, 4 mice were euthanized (Over anesthesia) to obtain footpad samples for subsequent analysis, including CFU counting and histopathological analysis.

### 2.11 Statistical analysis

Data are presented as mean ± standard deviation (SD). Statistical significance was analyzed by one-way ANOVA and Unpaired Student’s t-test with two-tailed P-values (95% CI). In all tests, P-values less than 0.05 were considered statistically significant.

## 3. Results

### 3.1. Deletion of *cnaA* or *cnaB* inhibits growth and alters morphological development in *F.monophora*

**Fig 1:**
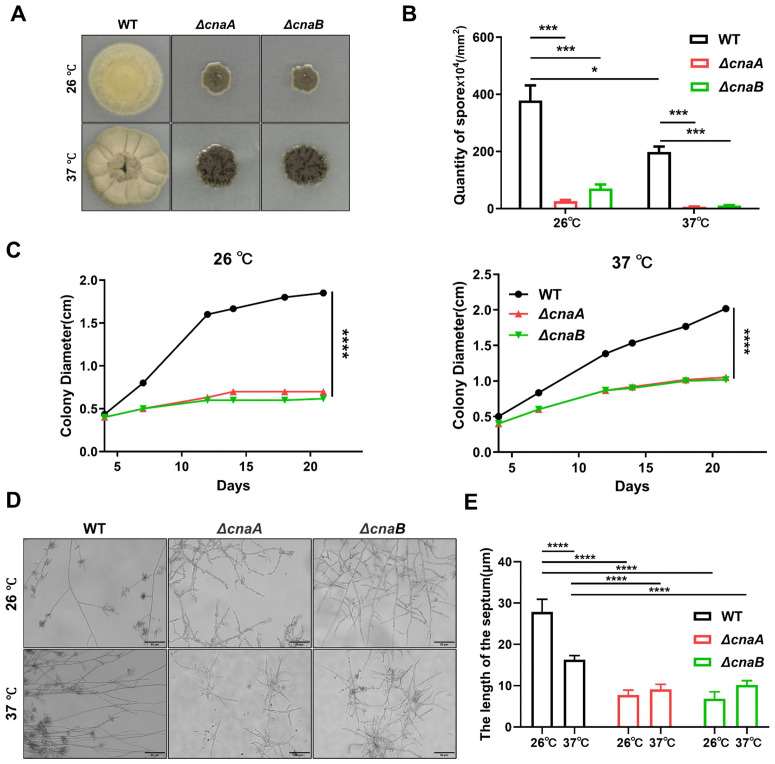
Effects of *cnaA* and *cnaB* deletion on the radial growth, spore quantity, and morphological development of *F. monophora.* **(A)** Colony morphology of the wild-type and mutant strains cultured at different temperatures for 14 days. The colony of the wild-type strain was dark green and villous, while the colonies of the mutant strains were black and villous free. **(B)** The number of collected spores of each strain at different temperatures. The mutant strains had fewer spores. n = 5. **(C)** Colony growth diameters of all strains at 26 °C and 37 °C. The mutant strains grow more slowly. n = 3. **(D)** Microscopic morphology of the wild-type and mutant strains. Mycelial swelling in mutant strains compared to wild-type strain. **(E)** Compartment length of the all strains at 26 °C and 37 °C. Shorter mycelial compartment length in mutant strains compared to wild-type strain. n = 5. The statistical analysis was performed using one-way ANOVA and Unpaired Student’s t-test (**, P < 0.01; ***, P < 0.001; ****, P < 0.0001). All comparisons were made relative to the wild-type strain group.

To investigate the roles of *cnaA* and *cnaB* in the growth and morphological development of *F. monophora*, deletion strains (*ΔcnaA* and *ΔcnaB*) were constructed and analyzed. The mRNA expression levels of the corresponding genes in the *cnaA* and *cnaB* gene mutation strains are undetectable (**S1 Fig**). Conidia were inoculated on PDA medium and incubated at 26 °C and 37 °C for 14 days. The wild-type formed dark green, villous colonies, while mutants produced black, non-villous colonies (**Fig 1A**). Mutants showed reduced spore production, especially at 37 °C (**Fig 1B**), slower growth rates (**Fig 1C**), and restricted mycelial and conidiophore development (**Fig 1D and 1E**). The compartment in fungal hyphae is associated with hyphal extension, and a longer compartment length contributes to hyphal invasion and expansion [22–24]. Proper separation and compartment length in fungi are critical for their growth, morphology, and function [22,25]. Compared to the wild-type strain, the mutant strains exhibit shorter mycelial compartment length, indicating irregular septation, which may correlate with growth constraints in the mutants and could lead to reduced virulence. To determine whether calcineurin deletion affects the transition of *F. monophora* to muriform cells, each strain was cultured with ATCC830 medium containing 0.1 mM CaCl_2_ (pH 2.5) for 60 days. In calcineurin-deficient strains, muriform cell formation was impaired in ATCC830 medium with 0.1 mM CaCl₂ (pH 2.5), indicating calcineurin’s role in calcium-mediated development (**S2 Fig**). The ultrastructural changes on mycelium and spore in two mutants were further observed using TEM and SEM. TEM and SEM analyses revealed that mutants had intact cell walls and organelles, with spore shapes appearing round and swollen at 26 °C, partially recovering at 37 °C (**S3 Fig**). This phenotypic rescue may be associated with the activation of pathways that maintain cellular morphology under thermal stress or calcineurin-independent compensatory signaling.

### 3.2. Deletion of *cnaA* or *cnaB* increases the sensitivity of *F. monophora* to antifungal drug, salt and cell wall stresses

Antifungal drug sensitivity tests were performed to evaluate the impact of *cnaA* and *cnaB* gene deletion on the susceptibility of *F. monophora* to various antifungal drugs. The mutant strains showed significantly lower MIC values compared to the wild-type strain (**Table 1)**, particularly with a 10-fold reduction in sensitivity to Posaconazole, Voriconazole, and Itraconazole. At the tested concentration (8 mg/L), tacrolimus alone exhibited no antifungal activity (MIC of tacrolimus: 16 mg/L) but demonstrated synergistic effects when combined with itraconazole or terbinafine, inhibiting 50 strains of *Fonsecaea* spp. *in vitro* (**Table 2**). Compared to terbinafine (14% synergy), combination with itraconazole showed significantly higher synergy (75%), and the geometric mean MIC of itraconazole decreased by 10-fold when co-administered with tacrolimus. No antagonistic interactions were observed.

**Table 1:** MICs of antifungal drug against the wild-type, *ΔcnaA* and *ΔcnaB* strains of *F. monophora.*

Drug/Strain	WT	*ΔcnaA*	*ΔcnaB*
Terbinefin	0.5	0.03	0.12
Anifengin	>8	2	2
Micafengin	>8	>8	>8
Carpofungin	>8	1	1
5-Fluorocytosine	16	2	2
Posaconazole	0.06	0.008	0.008
Voriconazole	0.06	0.008	0.008
Itraconazole	0.12	0.015	0.015
Fluconazole	16	8	4
Amphotericin B	4	2	2

**Table 2:** The FICIs of combination of tacrolimus with itraconazole and terbinafine against different *Fonsecaea* spp.

Drug/Strain (n = 50)	Tacrolimus/Itraconazole (FICI)				Tacrolimus/Terbinafine (FICI)			
	≤0.5	>0.5 ~ 1	>1 ~ 2	>2	≤0.5	>0.5 ~ 1	>1 ~ 2	>2
*F. monophora*	23	6	1	0	5	2	23	0
*F. nubica*	13	6	0	0	2	5	12	0
*F. pedrosoi*	1	0	0	0	0	0	1	0
Sum	37	12	1	0	7	7	36	0
Ratio (%)	74	24	2	0	14	14	72	0

FICI ≤0.5, > 0.5 to 1, > 1–2, and >2 indicate synergistic, additive, irrelevant, and antagonistic effects, respectively.

**Fig 2:**
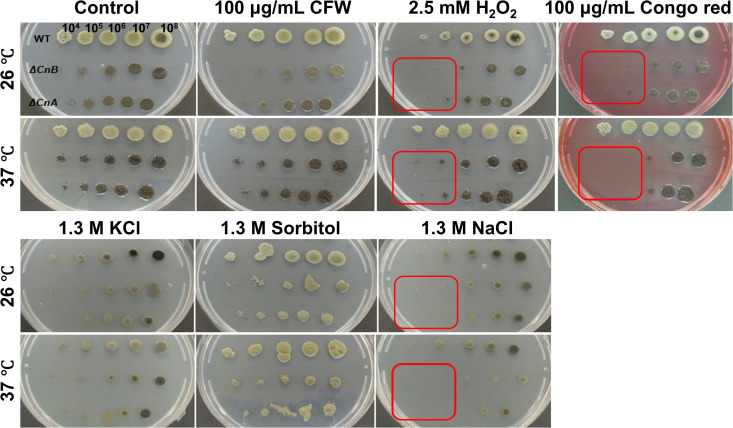
Involvement of the *cnaA* and *cnaB* in the stress response *in vitro* at 37 °C. Strains were cultivated on PDA medium supplemented with various chemical compounds at both 26 °C and 37 °C, as indicated in the images. The results demonstrated that H_2_O_2_, Congo red and NaCl significantly inhibited the growth of *ΔcnaA* and *ΔcnaB* mutants, while no notable effects were observed under other conditions: Calcofluor White (CFW), H_2_O_2_, KCl, and Sorbitol.

For stress resistance evaluation, the wild-type, *ΔcnaA*, and *ΔcnaB* strains were inoculated on PDA medium supplemented with various stressors (1.3 M KCl or NaCl for salt stress, 2.5 mM H₂O₂ for oxidative stress, 1.3 M sorbitol for osmotic stress, and 100 µg/mL Calcofluor White or Congo Red for cell wall stress). The strains were incubated at 26 °Cand 37 °C for 14 days. Results showed that H_2_O_2_, Congo Red and NaCl significantly inhibited the growth of *ΔcnaA* and *ΔcnaB* strains, indicating that calcineurin plays a role in oxidative stress, salt stress and cell wall stress adaptation (**Fig 2**). The oxidative stressor H₂O₂ generates reactive oxygen species (ROS) that cause severe cellular damage, including protein oxidation and DNA strand breaks. The hypersensitivity of the mutant strains to H₂O₂ suggests that calcineurin is crucial for managing oxidative damage. Similarly, Congo red inhibits fungal growth by binding to β-1,3-glucans in the cell wall and blocking polysaccharide synthesis [26], suggesting that the mutant strains may exhibit differences in the composition or structural organization of their cell wall compared to the wild-type strain (e.g., increased β-glucan exposure), rendering them more susceptible to growth inhibition.

### 3.3. Deletion of *cnaA* or *cnaB* enhances phagocytotic killing of *F. monophora*

**Fig 3:**
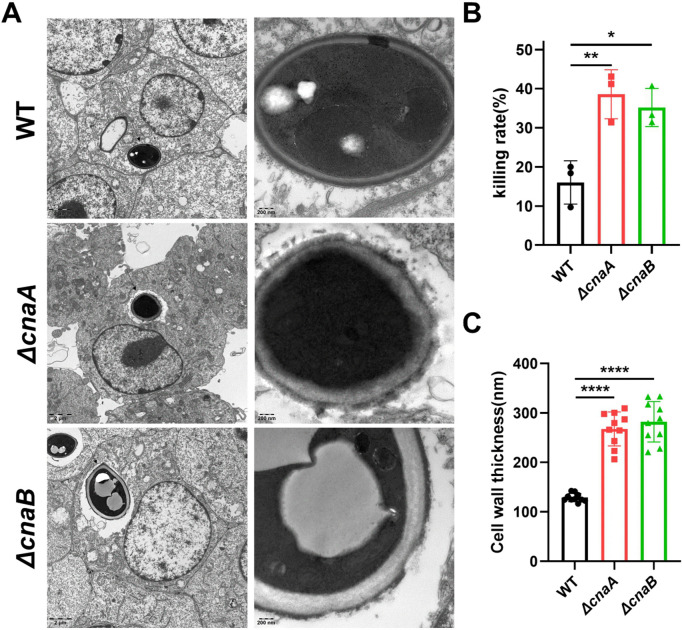
Requirement of *cnaA*/B for survival in macrophages. **(A)** After co-culturing with RAW264.7 macrophages for 24 h, TEM analysis was performed on each group. The wild-type strain maintained the integrity of the conidial cell wall and exhibited a uniform cytoplasm. In contrast, the conidia of the *ΔcnaA* and *ΔcnaB* mutants were engulfed by phagocytic vesicles (left side, black arrow), displaying cell wall defects (right side, black arrow). **(B)** The cell wall thickness of the conidia inside macrophages was measured for each group using Image **J.** The *ΔcnaA and* Δ*cnaB* strains exhibited thicker cell walls than that of the wild-type strain. n = 10. **(C)** Conidial survival was measured by CFU counting on PDA after lysing the infected macrophages. The CFU counts of the *ΔcnaA and* Δ*cnaB* strain were significantly lower than that of the wild-type strain. n = 3. The statistical analysis was performed using one-way ANOVA (**, P < 0.01). All comparisons were made relative to the wild-type strain group.

The impact of morphological changes and reduced growth rate in *ΔcnaA* and *ΔcnaB* mutants on immune killing was assessed by co-culturing all strains with RAW264.7 macrophages for 24 hours. TEM analysis showed that the wild-type strain maintained an intact cell wall with uniform cytoplasm, while *ΔcnaA* and *ΔcnaB* conidia were engulfed by phagocytic vesicles (left side, black arrow) with disrupted cell walls (right side, black arrow) (**Fig 3A**). Cell wall thickness measurements (via ImageJ) showed thicker walls in mutants compared to the wild type (**Fig 3B**). After 24 hours, macrophages infected with each strain were lysed to determine fungal load. The viable colony counts (CFUs) from mutant-infected macrophages were significantly lower after 7 days on PDA at 26 °C compared to wild-type-infected macrophages (**Fig 3C**). These data collectively suggest that the disruption of calcineurin signaling (in Δ*cnaA* and Δ*cnaB* mutants) significantly enhances their susceptibility to macrophage-mediated killing, likely due to critical alterations in cell wall integrity and overall structural stability within macrophages.

### 3.4. Deletion of *cnaA* or *cnaB* attenuates *F. monophora* virulence

**Fig 4:**
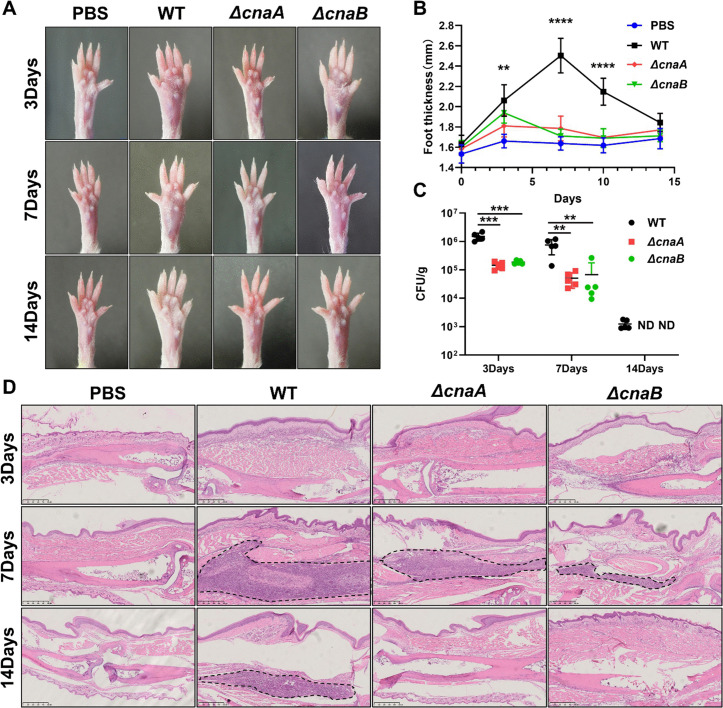
Effect of *cnaA* and *cnaB* deletion on virulence in the *F. monophora* infection mouse model. **(A)** Time-scaled photograph of representative mice footpad infected with wild-type, *ΔcnaA* and Δ*cnaB*, respectively, at 3, 7, and 14 dpi (days post infection). **(B)** Foot thickness measurements of mice infected with different strains over time. The area enclosed by the dotted line is the infiltration area of inflammatory cells. **(C)** Fungal load in the footpads of infected mice at various dpi. CFU counts for the *ΔcnaA* and Δ*cnaB* strains were significantly lower compared to the wild-type strain. **(D)** Histopathological analysis of footpads injected with all strains at different time points (day 3, 7, and 14). Compared to footpads injected with the wild-type strain, those injected with the *ΔcnaA* and Δ*cnaB* exhibited less swelling, a significantly reduced number of CFUs in the tissues, fewer inflammatory cells in infected tissues. The black dashed line delineates the boundary of inflammatory infiltration. n = 5. All statistical analysis were performed using two-tailed t-test, and the results were statistically significant (*, P < 0.05; ***, P < 0.001; ****, P < 0.0001). All comparisons were made relative to the wild-type strain group.

The virulence of *ΔcnaA* and *ΔcnaB* mutants was tested in a murine paw infection model. Both mutants showed significantly lower virulence than the wild-type strain, with less severe paw swelling and inflammation (**Fig 4A**, **4B and 4C**). Infected footpads of mutant-infected mice had lower fungal burdens that decreased over time (**Fig 4C**). Pathological examination revealed modest inflammation in mutant-infected footpads compared to extensive inflammation in wild-type-infected footpads (**Fig 4B**). In the *G. mellonella* larvae model, survival rates were higher for larvae infected with mutants, and fungal burdens were lower and less variable compared to the wild-type strain (**S4 Fig**). These data collectively demonstrate that calcineurin is essential for the full virulence of *F. monophora* in vivo. The attenuated pathogenicity observed in both mammalian and invertebrate infection models strongly suggests that calcineurin signaling plays a conserved and critical role in enabling the fungus to establish and maintain infection across different host environments.

### 3.5. Complementation of *cnaA* or *cnaB* rescues mutant phenotypes

**Fig 5:**
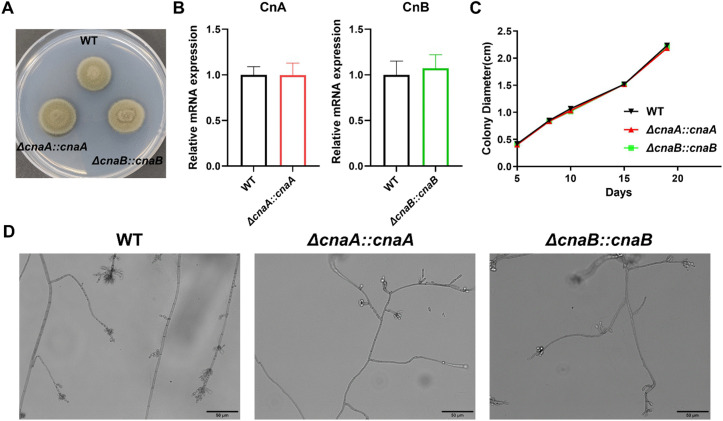
Growth and morphological restoration with reconstituted *cnaA* and *cnaB.* After gene complement, both growth rate and morphology were restored. **(A)** Colony morphology of wild-type and complemented strains. **(B)** The mRNA expression of *cnaA* and *cnaB* gene in wild-type and complemented strains. n = 3. **(C)** Colony growth diameter for all strains. n = 3. **(D)** Microscopic morphology of wild-type and complemented strains.

To confirm the phenotypic changes observed in the mutants, we constructed reconstituted strains with *cnaA* and *cnaB*. The results showed that the colony morphology of each complemented strain was identical to that of the wild-type strain, appearing dark green and fluffy (**Fig 5A**). The expression levels of can and *cnaB* genes in the complemented strains were the same as that of the wild-type strain, indicating successful complementation (**Fig 5B**). The growth rate of the complemented strains was also fully restored (**Fig 5C**) and their micromorphology was indistinguishable from of the wild-type strain (**Fig 5D**). These data provide critical genetic evidence that the phenotypic defects observed in the Δ*cnaA* and Δ*cnaB* mutants are specifically due to the disruption of the calcineurin genes, rather than secondary mutations or off-target effects.

### 3.6. *crzA* knockout does not replicate the phenotypes of *ΔcnaA* and *ΔcnaB*

**Fig 6:**
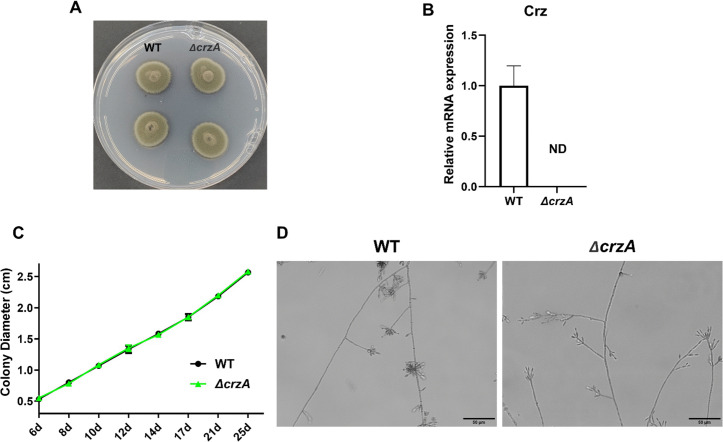
Morphology and growth rate were not affected in *crzA* gene knockout strain. **(A)** Colony morphology of the *ΔcrzA* strain was identical to that of the wild-type. **(B)** The mRNA expression of *crzA* gene mutant was undetectable following knockout. n = 3. **(C)** Colony growth diameter of the *ΔcrzA* mutant remained a comparable rate to the wild-type strain. n = 4. **(D)** Microscopic morphology exhibited no significant difference between the *ΔcrzA* mutant and the wild-type strain. The statistical analysis was performed using one-way ANOVA, and the results were statistically significant (**, P < 0.01; ***, P < 0.001). All comparisons were made relative to the wild-type strain group.

The protein sequence alignment with the *Aspergillus fumigatus* putative C2H2 transcription factor Crz1 (*crzA*) gene (Afu1g06900) revealed that AYO21_03504 exhibits the highest homology, with a Query Cover of 79% and a sequence identity of 48.66%, and it contains a C2H2 zinc finger structural motif. All other aligned genes showed Query Cover values below 17%, supporting the designation of AYO21_03504 as the putative *crzA* gene in *F. monophora*. The *crzA* knockout did not alter the colony morphology, which remained dark green and fluffy, similar to the wild-type strain (**Fig 6A**). Successful *crzA* gene knockout was confirmed by the absence of *crzA* expression (**Fig 6B**). The growth rate of the *crzA* knockout strain was comparable to that of the wild-type strain (**Fig 6C**), and its micromorphology showed no noticeable difference from the wild-type strain (**Fig 6D**). These findings demonstrate that *crzA* is not the essential downstream factor mediating the phenotypic changes in *ΔcnaA* and *ΔcnaB* knockout strains in *F. monophora*.

## 4. Discussion

Calcineurin is a widely conserved serine-threonine-specific protein phosphatase in eukaryotes, initially identified as a calcium- and calmodulin-binding protein [27]. Calcineurin is a heterodimer composed of a catalytic A subunit (*cnaA*) and a regulatory B subunit (*cnaB*), working together to dephosphorylate nuclear factor of activated T cells (NFAT) and facilitate its nuclear translocation [28]. It plays a central role in growth, ion homeostasis, and stress responses in fungal pathogens [29]. The calcineurin signaling pathway regulates genes linked to cell wall synthesis; and it is involved for the formation of infectious structures, intra-host propagation, and the dimorphic transition (yeast-to-mycelial) observed in various pathogenic fungi [27]. Our study significantly advances this understanding by systematically linking calcineurin function to morphological development, stress adaptation, and virulence in *F. monophora*, a major agent of chromoblastomycosis, thereby establishing it as a multifaceted therapeutic target.

In *Penicillium marneffei*, calcineurin-deficient strains show impaired conidiophore development, reduced germination and production of conidia, and defects in mycelial and yeast morphogenesis [30]. These findings align with results in *F. monophora* in this study, where calcineurin deficiency led to reduced spore production and significant changes in colony morphology and ultrastructure, suggesting that the calcineurin signaling pathway serves as a key regulator for hyphae and spore formation.

In fission yeast, calcineurin deletion disrupts Rho1-GTPase -mediated glucan synthesis, resulting in pronounced cell wall defects [31]. Similarly, *Botrytis cinerea* calcineurin-deficient strain displays comparable cell wall defects and loss of membrane integrity, which slows the penetration of conidia and hyphae into leaf tissues and weakens the response to cation stress [32]. In *A. fumigatus,* deletion of a single calcineurin A or B subunit or both subunits results in defects in mycelial growth, conidial germination, stress adaptation, and cell wall integrity, ultimately impairing virulence [33]. Calcineurin signaling in *Mucor circinelloides* coordinates the yeast-mycelial transition and regulates spore size, which are crucial for pathogenicity and host-pathogen interactions, including phagolysosome maturation and host cell death [34]. In *F. monophora*, however, while calcineurin-deficient strains showed altered spore and hyphal morphology (producing rounded spores and notably swollen hyphae), their stress response to external stimuli remained largely unchanged. However, calcineurin-deficient mutants did not convert to muriform cells *in vitro* as observed in the wild-type strain, suggesting the importance of calcineurin in muriform cells transformation.

The role of calcineurin in fungal virulence has been well-documented in both plant and human pathogenic fungi. For instance, the study on *A. fumigatus* have shown that deletion of the catalytic subunit A of calcineurin results in reduced virulence in animal models, with lower fungal loads in lung tissues compared to the wild-type strain [35]. In *Ustilago maydis*, calcineurin signaling is essential for adaptation to various environmental stresses, cell wall integrity and virulence; deletion of the catalytic subunit of calcineurin impacts sexual reproduction [36]. In this study, consistent with the morphological changes, macrophages exhibited enhanced killing efficacy against the calcineurin mutant strains (Fig 3). This may result from the mutant’s failure to adapt to intracellular stress, impaired cell wall integrity and morphogenesis, and inability to block phagosome maturation and lysosomal fusion, collectively leading to reduced survival within macrophages [30,37]. In animal infection model, the calcineurin mutant strains showed an attenuated inflammatory response and reduced virulence (Fig 4), likely due to alterations in cell wall composition (e.g., increased β-glucan exposure) or growth defects, which enhances recognition and clearance by the host innate immune system [38,39]. Specifically, macrophages and neutrophils demonstrated enhanced phagosome maturation and cytokine production, facilitating immune-mediated fungal clearance. Together, these findings emphasize that the calcineurin pathway is required for sustaining fungal virulence by modulating host immune responses and antimicrobial defenses.

*crzA* is a well-studied downstream transcription factor of calcineurin [40], and serves as the fungal counterpart to mammalian NFAT [41]. Calcineurin activates downstream responses by dephosphorylating *crzA*, facilitating its nuclear translocation. Although *crzA* and its homologs are crucial CN targets in many fungi, *crzA* mutant strains typically exhibit milder virulence or phenotypic abnormalities compared to CN mutant strains. Interestingly, *crzA* deletion impacts fungal species differently [42,43]. For example, in *A. fumigatus* (where Crz1 is termed *crzA*), *crzA* deletion causes growth, conidiation and virulence defects [44], whereas in *Candida albicans* (*C. albicans*), deletion of *crzA* affects pH sensitivity, mycelial growth and antifungal drug resistance without affecting virulence [45,46]. Although *crzA* and its homologous genes are key targets of calcineurin in many fungi, *crzA* mutant strains exhibit only mild impacts on virulence, indicating that calcineurin coordinates cellular functions through *crzA*-independent pathways. To date, putative calcineurin targets involved in calcineurin-*crzA*-independent pathways have been identified in two model organisms: *Saccharomyces cerevisiae* and *Cryptococcus neoformans* [27]. In this study, we identified the *F. monophora crzA* gene (AYO21_03504) by BLAST protein sequence analysis. Deletion of *crzA* did not significantly affect colony morphology, hyphae, conidia formation, or growth rate, suggesting that the phenotypic changes observed from calcineurin deletion in *F. monophora* are independent of *crzA*. These findings imply that other uncharacterized calcineurin substrates may play a critical role in mediating these effects. While multi-omics techniques (e.g., transcriptomics and proteomics) would be required to definitively identify these alternative pathways (a key direction for future research), our genetic evidence firmly establishes the existence of a *crzA*-independent signaling axis governing key virulence traits in *F. monophora*.

Our data indicate that calcineurin regulates antifungal drug sensitivity in this organism. Previous studies have demonstrated that combining calcineurin inhibitors with known antifungal compounds synergistically inhibits the growth of drug-resistant fungal strains [47]. Association of calcineurin with antifungal resistance has been demonstrated in both *C. albicans* [48] and *A. fumigatus* [49]. Consistent with these findings, our study revealed that calcineurin knockout strains of *F. monophora* exhibit increased susceptibility to antifungal drugs. Tacrolimus (FK506), a calcineurin inhibitor and a natural fungal product, is primarily used as an immunosuppressant to inhibit signaling events required for T cell activation and calcium signaling, making it valuable in preventing organ rejection [50]. Studies have reported that tacrolimus demonstrates synergistic effects when combined with antifungal agents. For instance, its combination with caspofungin has shown synergistic or additive interactions against *A. fumigatus* [51] and azole-resistant *Candida* species [52,53]. In our *in vitro* analyses of *F. monophora,* we evaluated the antifungal effects of tacrolimus alone and in combination with itraconazole and terbinafine. We found a stronger synergistic antifungal effect with tacrolimus and itraconazole, as 74% of clinical isolates showed a FICI ≤0.5, providing theoretical support for this combination in clinical CBM therapy. However, we acknowledge the clinical challenge of systemic immunosuppression by tacrolimus. Therefore, future therapeutic development should focus on strategies that circumvent this limitation, such as topical application for localized infections or the exploration of novel, fungal-selective calcineurin inhibitors, which our study strongly rationalizes.

In summary, our research demonstrated that calcineurin deficiency markedly reduces spore yield, growth rate, morphology, and virulence in *F. monochora*. Meanwhile, compared to the wild-type strain, calcineurin mutants showed increased sensitivity to oxidative stress, salt stress, cell wall stress and antifungal drugs; however, these phenotypic changes were independent of the transcription factor *crzA*, as the deletion of *crzA* did not significantly cause the same phenotypic changes. While our findings contribute to understand the pathological mechanism of *F. monophora,* further studies are necessary to fully elucidate the roles of the virulence factors and their therapeutic potentials*.*

## Supporting information

S1 Fig The mRNA expression levels of *cna*A and *cna*B genes in wild-type and mutant strains (ND means not detected). n = 3. (DOCX)

S2 Fig Morphological analysis of muriform cell transition in each strain cultured in ATCC830 medium supplemented with 0.1mM CaCl_2_ (pH 2.5) for 60 days. The black arrow indicates the muriform cell.
(DOCX)

S3 Fig The spore and mycelium structure were changed in ∆*cnaA* and ∆*cnaB* mutants. (A) Internal fungal structures were observed by TEM after 14 days of growth on PDA medium at 26 °C and 37 °C. All strains exhibited smooth, intact cell walls with clearly visible organelles, suggesting a mild effect of *cnaA* and *cnaB* on the cellular organelles of *F. monophora*. (B) Morphological changes were examined by SEM after 14 days of growth on PDA media at 26 °C and 37 °C. SEM images showed that the wild- type strain produced smooth, oval spores, whereas both mutant strains produced smooth, round spores with significantly swollen mycelium at 26 °C, although this effect was less pronounced at 37 °C.
(DOCX)

S4 Fig Virulence of *cnaA* and *cnaB* mutants in the *G. mellonella* larvae infection model. (A) Survival rates of *G. mellonella* larvae infected with all strains. n = 25. (B) Histological analysis of infected tissue of *G. mellonella* for each strain. The larvae were fixed, embedded in paraffin and stained with HE. The black areas indicate mycelium spreading through the larval tissue. (C) CFU counts showing that the fungal burden in the wild-type strain is higher and increases during infection, while changes in mutant strains are not significant. n = 4. Statistical analyses were performed using a two-tailed t-test, with results indicating significance (*, P < 0.05). All comparisons were made relative to the wild-type strain group.
(DOCX)

S1 Table Strains and plasmids used in this study. (DOCX)

S2 Table Primers used in this study. (DOCX)
