# Correction: Calcineurin regulates morphological development, stress responses and virulence in *Fonsecaea monophora*

**DOI:** 10.1371/journal.pntd.0014216

**Published:** 2026-04-16

**Authors:** Minying Li, Huan Huang, Dongmei Li, Judun Zheng, Yinghui Liu, Yangxia Chen, Zhenmou Xie, Liyan Xi, Hongfang Liu

Figs 2 and 3 were uploaded incorrectly. Please see the correct Figs 2 and 3 here.

**Fig 2:**
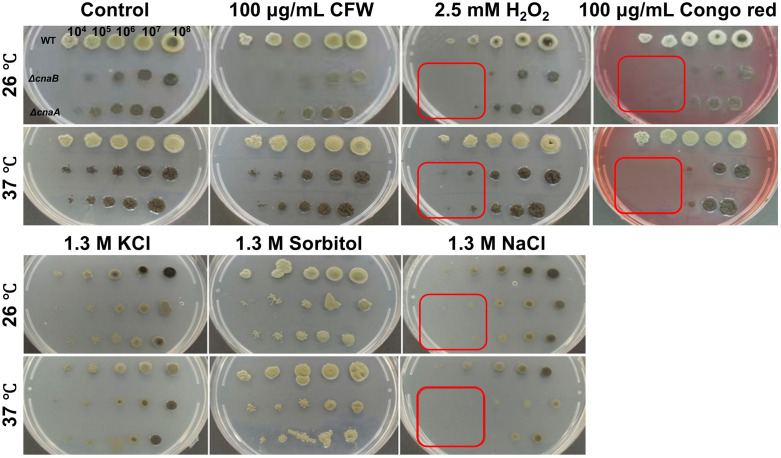
Involvement of the *cnaA* and *cnaB* in the stress response *in vitro* at 37 °C. Strains were cultivated on PDA medium supplemented with various chemical compounds at both 26 °C and 37 °C, as indicated in the images. The results demonstrated that H_2_O_2_, Congo red and NaCl significantly inhibited the growth of *ΔcnaA* and *ΔcnaB* mutants, while no notable effects were observed under other conditions: Calcofluor White (CFW), H_2_O_2_, KCl, and Sorbitol.

**Fig 3:**
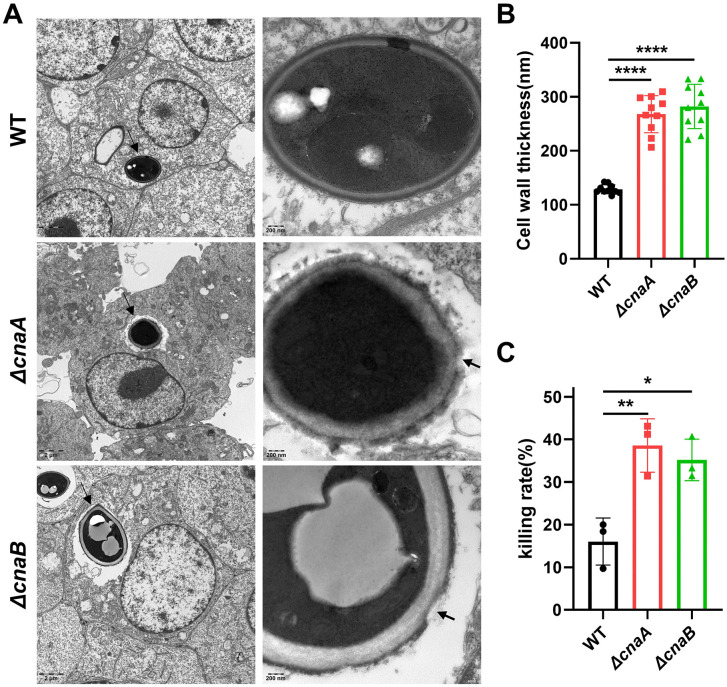
Requirement of *cnaA*/B for survival in macrophages. (A) After co-culturing with RAW264.7 macrophages for 24 h, TEM analysis was performed on each group. The wild-type strain maintained the integrity of the conidial cell wall and exhibited a uniform cytoplasm. In contrast, the conidia of the *ΔcnaA* and *ΔcnaB* mutants were engulfed by phagocytic vesicles (left side, black arrow), displaying cell wall defects (right side, black arrow). (B) The cell wall thickness of the conidia inside macrophages was measured for each group using Image J. The *ΔcnaA and* Δ*cnaB* strains exhibited thicker cell walls than that of the wild-type strain. n = 10. (C) Conidial survival was measured by CFU counting on PDA after lysing the infected macrophages. The CFU counts of the *ΔcnaA and* Δ*cnaB* strain were significantly lower than that of the wild-type strain. n = 3. The statistical analysis was performed using one-way ANOVA (**, P < 0.01). All comparisons were made relative to the wild-type strain group.
