# Supplementary material for: Calcineurin regulates morphological development, stress responses and virulence in Fonsecaea monophora
Source: PLoS Negl Trop Dis. 2025 Dec 10;19(12):e0013816. doi: 10.1371/journal.pntd.0013816 (PMC12711089; doi:10.1371/journal.pntd.0013816)
Supplement: S1 Fig — (DOCX) [file pntd.0013816.s001.docx]

**
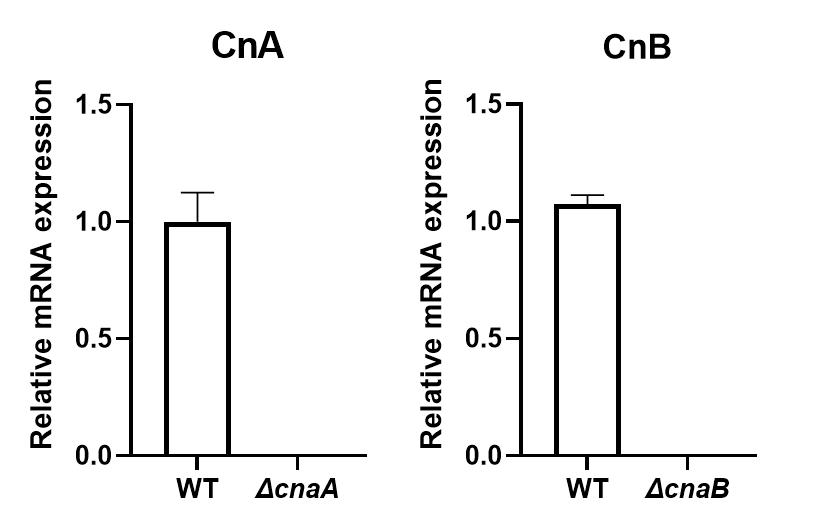
**

**S1 Fig**. The mRNA expression levels of *cnaA* and *cnaB* genes in wild-type and mutant strains (ND means not detected). n = 3.
