# Supplementary material for: Calcineurin regulates morphological development, stress responses and virulence in Fonsecaea monophora
Source: PLoS Negl Trop Dis. 2025 Dec 10;19(12):e0013816. doi: 10.1371/journal.pntd.0013816 (PMC12711089; doi:10.1371/journal.pntd.0013816)
Supplement: S2 Fig — The black arrow indicates the muriform cell. (DOCX) [file pntd.0013816.s002.docx]

**
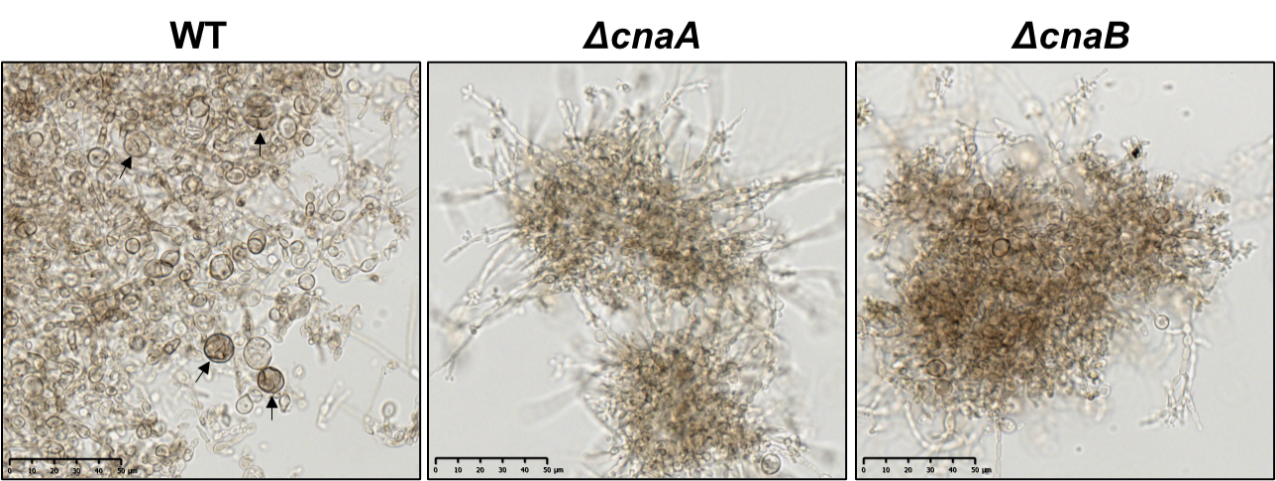
**

**S2 Fig.** Morphological analysis of muriform cell transition in each strain cultured in ATCC830 medium supplemented with 0.1mM CaCl_2_ (pH 2.5) for 60 days. The black arrow indicates the muriform cell.
