# Supplementary material for: Calcineurin regulates morphological development, stress responses and virulence in Fonsecaea monophora
Source: PLoS Negl Trop Dis. 2025 Dec 10;19(12):e0013816. doi: 10.1371/journal.pntd.0013816 (PMC12711089; doi:10.1371/journal.pntd.0013816)
Supplement: S3 Fig — (A) Internal fungal structures were observed by TEM after 14 days of growth on PDA medium at 26 °C and 37 °C. All strains exhibited smooth, intact cell walls with clearly visible organelles, suggesting a mild effect of cnaA and cnaB on the cellular organelles of F. monophora. (B) Morphological changes were examined by SEM after 14 days of growth on PDA media at 26 °C and 37 °C. SEM images showed that the wild- type strain produced smooth, oval spores, whereas both mutant strains produced smooth, round spores with significantly swollen mycelium at 26 °C, although this effect was less pronounced at 37 °C. (DOCX) [file pntd.0013816.s003.docx]

**
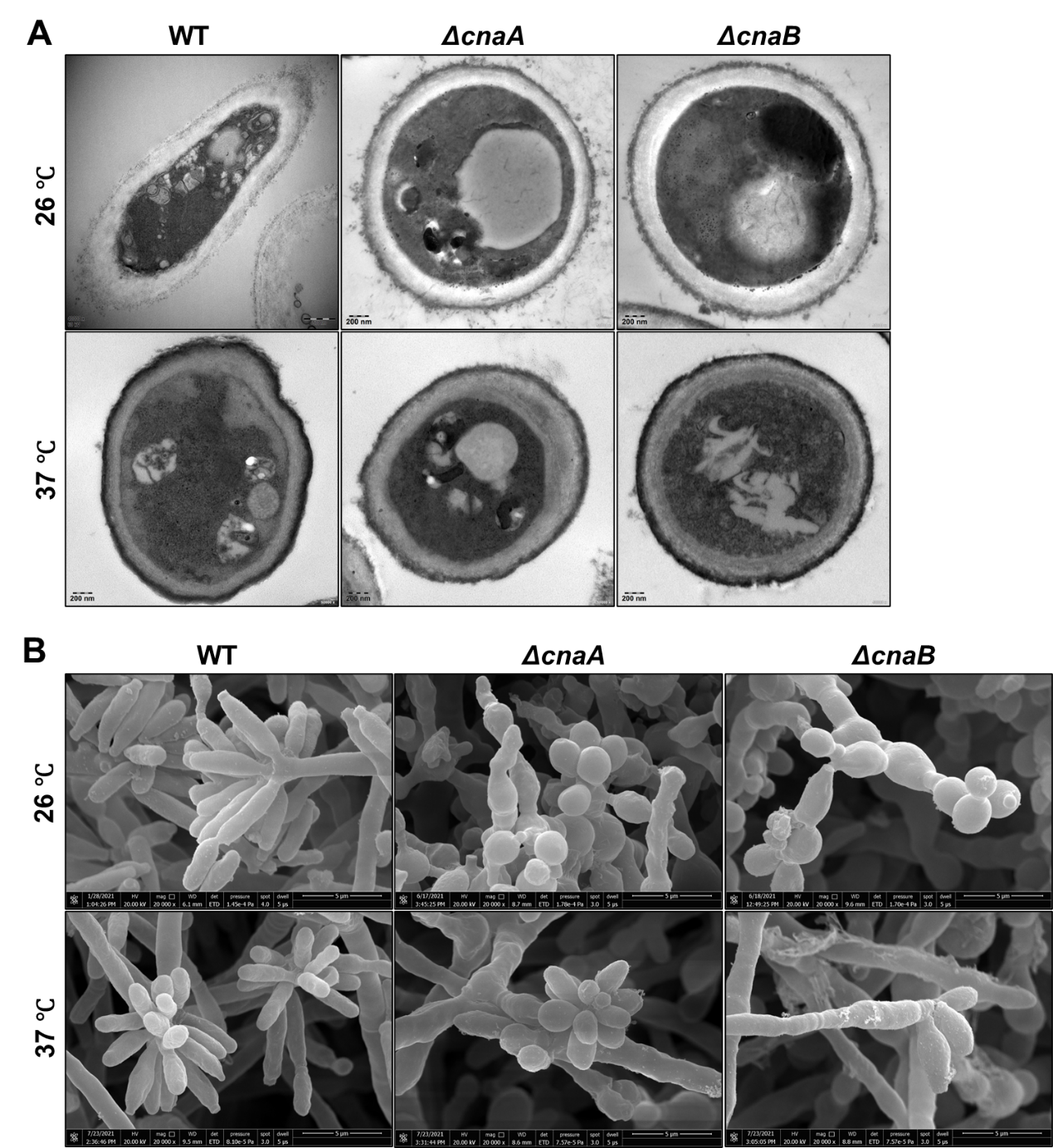
**

**S3 Fig.** The spore and mycelium structure were changed in *∆cnaA* and *∆cnaB* mutants. (A) Internal fungal structures were observed by TEM after 14 days of growth on PDA medium at 26 °C and 37 °C. All strains exhibited smooth, intact cell walls with clearly visible organelles, suggesting a mild effect of *cnaA* and *cnaB* on the cellular organelles of *F. monophora*. (B) Morphological changes were examined by SEM after 14 days of growth on PDA media at 26 °C and 37 °C. SEM images showed that the wild- type strain produced smooth, oval spores, whereas both mutant strains produced smooth, round spores with significantly swollen mycelium at 26 °C, although this effect was less pronounced at 37 °C.
