# Supplementary material for: Calcineurin regulates morphological development, stress responses and virulence in Fonsecaea monophora
Source: PLoS Negl Trop Dis. 2025 Dec 10;19(12):e0013816. doi: 10.1371/journal.pntd.0013816 (PMC12711089; doi:10.1371/journal.pntd.0013816)
Supplement: S4 Fig — (A) Survival rates of G. mellonella larvae infected with all strains. n = 25. (B) Histological analysis of infected tissue of G. mellonella for each strain. The larvae were fixed, embedded in paraffin and stained with HE. The black areas indicate mycelium spreading through the larval tissue. (C) CFU counts showing that the fungal burden in the wild-type strain is higher and increases during infection, while changes in mutant strains are not significant. n = 4. Statistical analyses were performed using a two-tailed t-test, with results indicating significance (*, P < 0.05). All comparisons were made relative to the wild-type strain group. (DOCX) [file pntd.0013816.s004.docx]

**
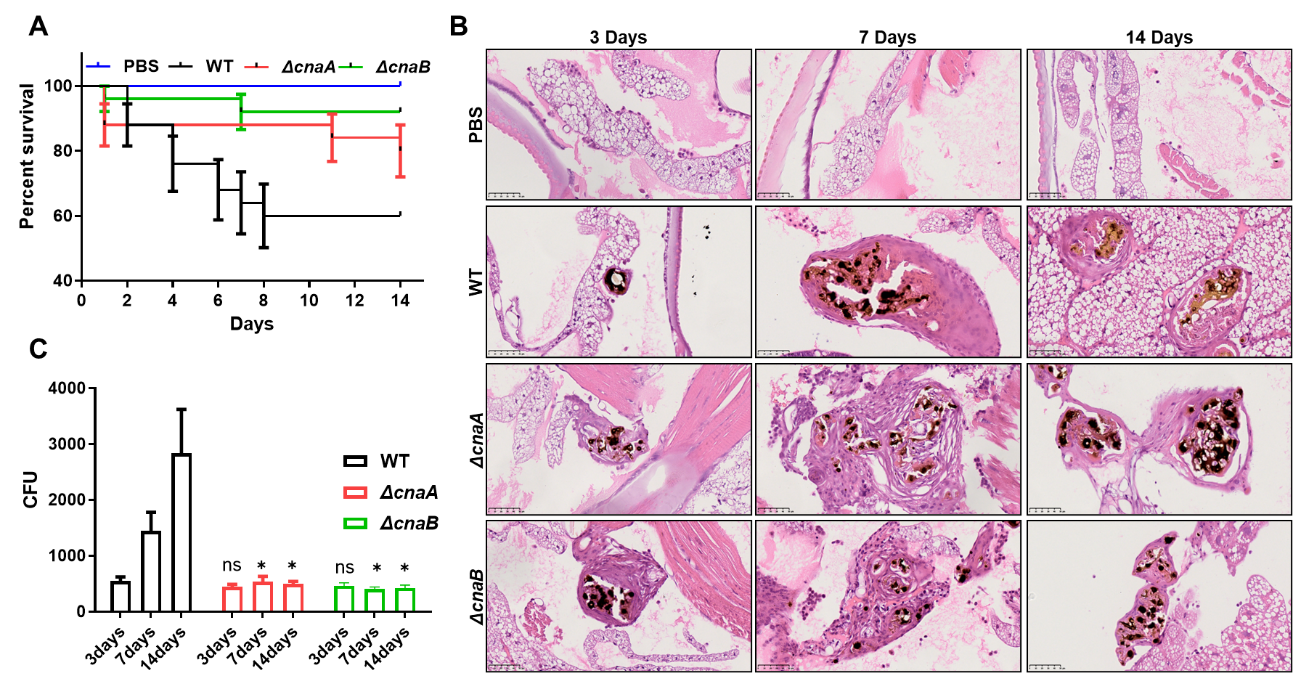
**

**S4 Fig.** Virulence of *cnaA* and *cnaB* mutants in the *G. mellonella* larvae infection model. (A) Survival rates of *G. mellonella* larvae infected with all strains. n = 25. (B) Histological analysis of infected tissue of *G. mellonella* for each strain. The larvae were fixed, embedded in paraffin and stained with HE. The black areas indicate mycelium spreading through the larval tissue. (C) CFU counts showing that the fungal burden in the wild-type strain is higher and increases during infection, while changes in mutant strains are not significant. n = 4. Statistical analyses were performed using a two-tailed t-test, with results indicating significance (*, P<0.05). All comparisons were made relative to the wild-type strain group.
