# Supplementary material for: Calcineurin regulates morphological development, stress responses and virulence in Fonsecaea monophora
Source: PLoS Negl Trop Dis. 2025 Dec 10;19(12):e0013816. doi: 10.1371/journal.pntd.0013816 (PMC12711089; doi:10.1371/journal.pntd.0013816)
Supplement: S1 Table — (DOCX) [file pntd.0013816.s005.docx]

**S1** **Table** Strains and plasmids used in this study.

| Strain or plasmid | Genotype or characteristic |
| --- | --- |
| CBS269.37 | Wild-type of *F. monophora* |
| *ΔcnaA* | Knock out *cnaA* of *F. monophora* |
| *ΔcnaB* | Knock out *cnaB* of *F. monophora* |
| *ΔcrzA* | Knock out *crzA* of *F. monophora* |
| *ΔcnaA::cnaA* | Complemented strain of *ΔcnaA* |
| *ΔcnaB::cnaB* | Complemented strain of *ΔcnaB* |
| *cnaA*-hph-pBHt2 | Plasmid containing the *cnaA* gene and hygromycin  resistance gene |
| *cnaB*-hph-pBHt2 | Plasmid containing the *cnaB* gene and hygromycin  resistance gene |
| *crzA*-hph-pBHt2 | Plasmid containing the *crzA* gene and hygromycin  resistance gene |
| *cnaA*-C-neo-pBHt2 | Plasmid containing the *cnaA* gene complemented fragment and geneticin resistance gene |
| *cnaB*-C-neo-pBHt2 | Plasmid containing the *cnaB* gene complemented fragment and geneticin resistance gene |
