# Supplementary material for: Calcineurin regulates morphological development, stress responses and virulence in Fonsecaea monophora
Source: PLoS Negl Trop Dis. 2025 Dec 10;19(12):e0013816. doi: 10.1371/journal.pntd.0013816 (PMC12711089; doi:10.1371/journal.pntd.0013816)
Supplement: S2 Table — (DOCX) [file pntd.0013816.s006.docx]

**S2 Table** Primers used in this study.

| Primer Name | Sequence (5’-3’) |
| --- | --- |
| *cnaA* KO arm1-F | gtttttaatgCTCGATCCCCACCACGGAGCTTG |
| *cnaA* KO arm1-R | taattcagtaCTCGAGTCATTCACTCATTGCCATTCTATCAAT |
| cnaA KO arm2-F | acatgattacgaattCGTGATTGTGAGGTGCGCTC |
| cnaA KO arm2-R | cgactctagaggatcTTCTCTGCCAGATCTCGATCCTTGA |
| cnaB KO arm1-F | gtttttaatgCTCGAACAAGACGATATACTTATTCTCACCACG |
| cnaB KO arm1-R | taattcagtaCTCGAGGCTGGCATGGCG |
| cnaB KO arm2-F | acatgattacgaattGGAGGGCGATTGAGGGGTGTGAC |
| cnaB KO arm2-R | cgactctagaggatcTATCGTAGTCGGAAAGAGGATCAACTTGAT |
| cnaA-Com arm1-F | cggacgtttttaatgctcgagTCCCCACCACGGAGCTTG |
| cnaA-Com arm1-R | gcgttaattcagtaCTCGAGGTCATTCACTCATTGCCATT |
| cnaA-Com arm2-F | acatgattacgaattCTTGTCATCGTCGTCCTTGTAATCTTACAAGCTGAT  TCTCCTTG |
| cnaA-Com arm2-R | cgactctagaggatcTTCTCTGCCAGATCTCGATCCTTGA |
| crzA arm1-F | taattcagtaCTCGACTGAGGTTGTTTTTGACGTTCGTTTG |
| crzA arm1-R | gtttttaatgCTCGAAAACGAGACGAGTTTGTGTCAGTAATGAGCA |
| crzA arm2-F | acatgattacgaattCTTTTTCAACACAACCCACT |
| crzA arm2-R | cgactctagaggatcGTGGATTTGGGGGGGGTATGC |
| q-*cnaA*-F | CTGGGGGATTATGTCGACCG |
| q-*cnaA*-R | TGCATGCGTCGTAGACCTTT |
| q-*cnaB*-F | AACCCAACAATGCCGCTCTA |
| q-*cnaB*-R | GTTTTCGCAACCTGTCCACC |
| q-*crzA*-F | ACCGGACACAAGTAATGCGT |
| q-*crzA*-R | TACGGGGATTCCGAGAGGTT |
| q-18 s RNA-F | AACGAACGAGACCTTGACCT |
| q-18 s RNA-R | TTCGGCCAAGGTGATGTACT |
